# Sociodemographic characteristics of women participating to the LOVE-THEM (Listening to Obstetric Violence Experiences THrough Enunciations and Measurement) investigation in Italy

**DOI:** 10.1016/j.dib.2018.04.146

**Published:** 2018-05-08

**Authors:** Claudia Ravaldi, Elena Skoko, Alessandra Battisti, Michela Cericco, Alfredo Vannacci

**Affiliations:** a CiaoLapo Onlus, Charity for Stillbirth and Perinatal Grief Support, Prato, Italy; b Human Rights in Maternity and Childbirth Research Unit, Multimedia Lab for Comparative Law, Department of Political Sciences, "Roma Tre" University, Rome, Italy; c La Goccia Magica, Rome, Italy; d Department of Neurosciences, Psychology, Drug Research and Child Health, University of Florence, Florence, Italy

## Abstract

Data here reported are sample characteristics of the first nation-wide community based survey on 'obstetric violence' (OV) conducted in a high-income country (Italy). The initiative is the extension of the social media campaign “#Bastatacere: mothers have voice” that in 2016 put under national spotlight the hidden phenomenon of abuse and disrespect in childbirth in hospital facilities, advocating for a respectful maternity care. The questionnaire LOVE-THEM was firstly developed in an open format and then revised according to WHO definition of disrespect and abuse in childbirth, within human rights based approach. The survey was conducted through on line interviews (CAWI method, quota sampling) with 424 respondents representing a significant national sample of mothers with children aged 0–14 years. Here we report summary tables describing the sample distribution according to the socio-demographic characteristics (instruction, employment status, social and economic class), including the number and the age of children. The responding sample is proportionally appropriate and correctly representative of about 5 millions of childbearing women in Italy.

**Specifications Table**

**Table:** 

Subject area	*Medicine*
More specific subject area	*Obstetrics and perinatal psychology*
Type of data	*Tables, charts, and spreadsheet file*
How data was acquired	*Web based survey*
Data format	*Partially analyzed*
Experimental factors	*n.a.*
Experimental features	*n.a.*
Data source location	*Italy (nation-wide)*
Data accessibility	*Data is with this article*
Related research article	*Ravaldi C., Skoko E., Battisti A., Cericco M., Vannacci A., Abuse and disrespect in childbirth assistance in Italy: a community-based survey.* Eur J Obstet Gynecol Reprod Biol. 2018 May;224:208-209. 10.1016/j.ejogrb.2018.03.055.

**Value of the data**

• Many women across the globe experience disrespectful and abusive treatment during childbirth, conditions described as ‘obstetric violence’.
• Obstetric violence was first described and legally codified in Latin America, but its perception is increasing also in developed countries.
• Data here reported describe sociodemographic characteristics of the first nation-wide community based survey on obstetric violence conducted in a high-income country.
• Methods and data from this article may help researchers from all countries to conduct similar studies in their national settings.

## 1 Data

Data here reported are sample characteristics of the first nation-wide community based survey on OV conducted in a high-income country (Italy). The initiative is the extension of the social media campaign “#Bastatacere: mothers have voice” that in 2016 put under national spotlight the hidden phenomenon of abuse and disrespect in childbirth in hospital facilities, advocating for a respectful maternity care. The survey was conducted by means of a questionnaire firstly developed in an open format and then revised according to WHO definition of disrespect and abuse in childbirth, within human rights based approach [1].

## 2 Experimental design, materials, and methods

### 2.1 Sample

**Fig. 1:**
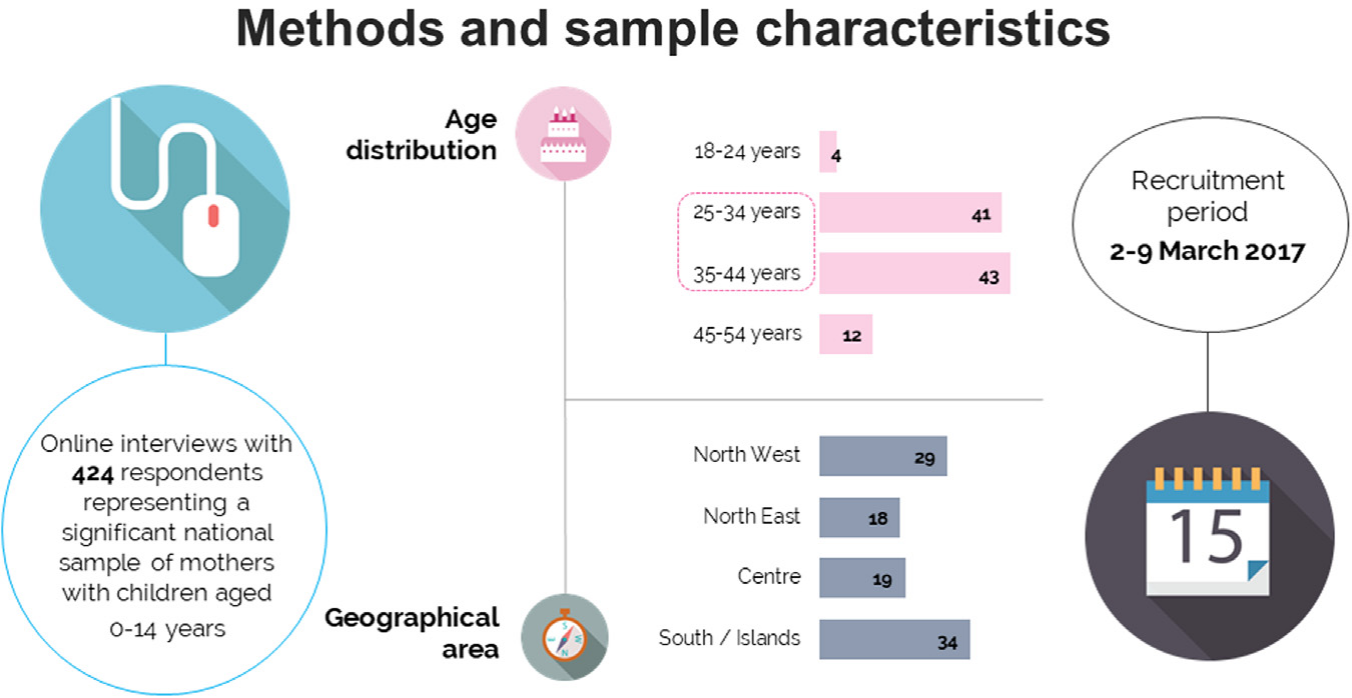
Methods and sample characteristics of the LOVE-THEM investigation.

The survey was conducted through on line interviews (CAWI method, quota sampling) with 424 respondents representing a significant national sample of mothers with children aged 0–14 years. The sample of interviewed women was selected with the participation of “Due punto zero research”, a society belonging to the Doxa Group, experienced in digital research. The starting sample targeted women of 20–60 years of age, according to the age percentage and geographical area. Random selection allowed for a reasonable distribution of the sample according to the socio-demographic characteristics (instruction, employment status, social and economic class), including the number and the age of children. The responding sample is proportionally appropriate and correctly representative of about 5 millions of childbearing women in Italy (Fig 1).

The “target universe”, female population within 20–60 years age range, was divided in two sections based on two characteristics: the age and the geographic area (North-West, North-East, Centre, South+Islands). The appropriate number of interviewees has been decided in order to be representative, faithfully reproducing the society in miniature scale.

The sample of 424 mothers can guarantee a minimum margin error of 4.8%, and a confidence level of 95%. The execution of the survey complied with the ASSIRM Professional Code of Conduct and with the international ESOMAR And MSPA codes, guaranteeing the methodological standards of the statistics and fulfilling the requirements of the applied scientific research. DOXA S.p.a is certified according to the international quality norm standards UNI EN ISO 9001:2008 for qualitative and quantitative market researches.

### 2.2 Questionnaire

The survey was conducted by means of the questionnaire LOVE-THEM (Listening to Obstetric Violence Experiences THrough Enunciations and Measurement), an instrument specifically developed for the project by the authors (AB, ES, CR and MC). LOVE-THEM was developed within the mainframe of the “#Bastatacere: mothers have voice” campaign, starting from open questions and then revising proposed items through a qualitative analysis conducted according to guidelines endorsed by FIGO and WHO for the study of abuse and disrespect during childbirth. LOVE-THEM, as used in the present research, is composed of two sections: section A explores sociodemographic features of women (data here reported, Table 1), section B explores several experiences, feelings and perceptions of women during childbirth - overall 37 closed-ended questions. The final version of the questionnaire, as well as a thorough methodological description, is under publication elsewhere (Table 1) (Fig. 1).

**Table 1:** Characteristics of the actual sample, conforming to the general distribution of the target population.

	**N (% out of 424)**
**Age range**	
20–24 years	15 (3.5%)
25–34 years	173 (40.8%)
35–44 years	183 (43.2%)
45–54 years	47 (11.1%)
55–60 years	6 (1.4%)
**Geographical area**	
North West	122 (28.8%)
North East	77 (18.2%)
Centre	82 (19.3%)
South / Islands	143 (33.7%)
**Education**	
Master/PHD	26 (6.1%)
University Degree/five years	64 (15.1%)
University Degree/Three years	71 (16.7%)
High School Degree	213 (50.2%)
Secondary School Degree	47 (11.1%)
Primary School Degree	2 (0.5%)
No school education	1 (0.2%)
**Number of children**	
1	169 (39.9%)
2	195 (46.0%)
3	50 (11.8%)
4	7 (1.7%)
5	3 (0.7%)
